# Dual Roles of Fer Kinase Are Required for Proper Hematopoiesis and Vascular Endothelium Organization during Zebrafish Development

**DOI:** 10.3390/biology6040040

**Published:** 2017-11-23

**Authors:** Emily M. Dunn, Elizabeth J. Billquist, Amy L. VanderStoep, Phillip G. Bax, Laura M. Westrate, Lisa K. McLellan, Shelby C. Peterson, Jeffrey P. MacKeigan, Aaron P. Putzke

**Affiliations:** 1 Department of Biology, Whitworth University, Spokane, WA 99251, USA; edunn@whitworth.edu (E.M.D.); pbax18@my.whitworth.edu (P.G.B.); 2 Department of Biology, Hope College, Holland, MI 49423, USA; ebillquist@luc.edu (E.J.B.); vande867@msu.edu (A.L.V.); lmclellan@wustl.edu (L.K.M.); scpete@umich.edu (S.C.P.); 3 Center for Cancer Cell Biology, Van Andel Research Institute, Grand Rapids, MI 49503, USA; laura.westrate@colorado.edu (L.M.W.); jeff.mackeigan@vai.org (J.P.M.)

**Keywords:** non-receptor tyrosine kinase, erythrocyte, vasculature, embryogenesis

## Abstract

Fer kinase, a protein involved in the regulation of cell-cell adhesion and proliferation, has been shown to be required during invertebrate development and has been implicated in leukemia, gastric cancer, and liver cancer. However, in vivo roles for Fer during vertebrate development have remained elusive. In this study, we bridge the gap between the invertebrate and vertebrate realms by showing that Fer kinase is required during zebrafish embryogenesis for normal hematopoiesis and vascular organization with distinct kinase dependent and independent functions. In situ hybridization, quantitative PCR and fluorescence activated cell sorting (FACS) analyses revealed an increase in both erythrocyte numbers and gene expression patterns as well as a decrease in the organization of vasculature endothelial cells. Furthermore, rescue experiments have shown that the regulation of hematopoietic proliferation is dependent on Fer kinase activity, while vascular organizing events only require Fer in a kinase-independent manner. Our data suggest a model in which separate kinase dependent and independent functions of Fer act in conjunction with Notch activity in a divergent manner for hematopoietic determination and vascular tissue organization.

## 1. Introduction

The formation of germ layers during vertebrate development leads to increasing levels of molecular fine tuning that result in the differentiation of specific tissue types. Proper differentiation relies heavily on transcriptional regulation and cellular adhesion, both of which are mediated by different types of kinase signaling. Kinase signaling during development has been well characterized and involves myriad kinases, including both receptor and non-receptor tyrosine kinases, such as the well-known Src family kinases (reviewed in [1,2,3]). Additionally, the emergence of the dualistic properties of some kinases has revealed novel avenues of protein functions which will further clarify complex signaling roles in cellular regulation.

One subset of non-receptor tyrosine kinases (NRTKs) is the Fes/Fps family that includes the Fer (Fes-Related) kinase. Fer kinase expression was first detected in both human and mouse (leukemic and erythroblast) cells [4], and has been shown to be highly conserved throughout evolution [5,6]. Fer kinase contains an N-terminal F-BAR domain, shown to be involved in stabilizing interactions with cytoskeletal elements that recruit Fer to the plasma membrane, a Src homology 2 (SH2) domain and a tyrosine kinase (TyrKin) domain [7]. Additionally, Fer also has a coiled-coil domain that facilitates homotypic oligomerization and autophosphorylation by forming homotrimers, which are not required for kinase activity [8]. Fer has been shown to be activated via pathways, such as epidermal growth factor receptor (EGFR) and platelet derived growth factor (PDGF), and phosphorylate protein targets, such as Stat3 [9,10,11,12,13,14]. However, the required in vivo functions of Fer during development remain largely unknown.

Fer kinase is conserved across invertebrate and vertebrate phyla. For example, Fer expression has been detected in sea sponges [15], nematodes [16], fruit flies [17,18], rats and humans [6]. In vertebrates, two versions of Fer have been observed in mice and humans, the full length version, found ubiquitously expressed, and a testis specific version, arising from an internal start site [6,19]. Interestingly, there is greater variability among invertebrates regarding different versions of Fer, such that while fruit flies have been shown to express both versions [18]; nematodes only express the truncated, but not the full length, version of Fer [16]. Recently, the full length version of Fer has been shown to be expressed during zebrafish development in a ubiquitous manner early, while later concentrating to the head region and fin buds [20]. In addition, the same study reported developmental defects ranging from convergent extension to craniofacial defects when knocking down Fer using morpholino technology [20].

Fer kinase has been linked to a variety of cellular functions, such as cytoskeletal interactions, mediating cadherin and integrin dependent adhesion that can lead to cellular migration [21,22,23]. Interestingly, the absence of Fer kinase has been shown to lead to developmental defects during embryonic enclosure in nematodes [16] and fruit flies [24], both of which are cadherin dependent adhesion functions of Fer. Fer kinase has also been shown to be involved in cellular proliferation under normal conditions in human and worms [25,26,27]. Importantly, Fer kinase has been reported to regulate cancer progression where it appears to be functioning in a finely tuned balance, such that when either over-expressed in renal cell carcinomas [28,29] or deleted in myeloid leukemia [30] or lung cancer [31], the cellular functions of Fer are perturbed and result in similar negative consequences (such as loss of cadherin-complex stability). Furthermore, an RNA interference screen effectively demonstrated that Fer kinase plays a significant role in cancer cell survival, and also that the loss of Fer enhanced cell death in conjunction with chemotherapeutic agents [32].

More recently, kinase inactive protein functions have been reported, showing that there are critical aspects of kinases that do not require their phosphorylation capacity to be intact (reviewed in [3]). Kinase independent roles for Fer have been demonstrated, due to the sustained viability and fertility of mice expressing a kinase inactive version of Fer [12], as well as the ability of a similar kinase inactivating mutations in Fer to rescue embryonic development in nematodes [16]. Interestingly, both Fer and Fes/Fps have been reported to be redundant in regulating hematopoiesis [33]. Parsing out the functions of Fer that are independent of kinase activity will shed more light on the complexities of the signaling pathways involved, and may allow for more clarification on overlapping roles within the Fes-Fps subfamily of NRTKs.

In this study, we characterized a novel expression pattern and functional component to Fer during zebrafish embryonic development, which has not previously reported. We report here that not only is Fer required for proper axis determination and formation of head structures during embryogenesis (as reported by [20], but also for proper organization of the vascular endothelium and hematopoietic proliferation in the trunk region. The absence of Fer results in disorganized trunk vasculature within the region of the cardinal vein (CV), dorsal aorta (DA) and intersegmental vessels (ISV), resulting in subsequent loss of erythrocyte circulation. Additionally, we observed an increase in hematopoietic cell markers in the region of the intermediate cell mass (ICM), corresponding to an increase in the number of erythrocytes. Finally, we demonstrated diverging roles for Fer, with Fer kinase activity required for the proper proliferation of hematopoietic cell populations, but a kinase independent role in proper organization of the vascular endothelium via the Notch pathway.

## 2. Materials and Methods

### 2.1. Transgenic Zebrafish Lines

Wildtype (AB) zebrafish as well as lines expressing either *fli1a::nEGFP (y7Tg)* or *gata1a::dsRed (sd2Tg)* were obtained from the Zebrafish International Resource Center (Eugene, OR, USA), from which we generated a stable *fli1a::nEGFP (y7Tg), gata1a::dsRed (sd2Tg)* double transgenic line. All lines were maintained according to standard zebrafish husbandry protocols, as previously described [34].

### 2.2. Fer Morpholino Knockdown

Antisense morpholino oligonucleotides (MOs) were generated by Gene Tools, LLC (Philomath, OR, USA). *fer*-MO1 (5′-CTGGAAGAGAGACAGAGATCACACT-3′) was designed against the splice acceptor site in intron 8 of *fer* in order to prevent proper splicing with exon 9, thus facilitating an exon-8–10 splice, generating a premature stop codon in the translated product. The control morpholino was a 25-mer of randomized nucleotides purchased from Gene Tools, LLC. The oligonucleotides were solubilized in RNase-free water at a concentration of 1 mM and stored according to the manufacturer’s instructions. The injections were made by diluting the oligonucleotides with phenol red and RNase-free water. Titration of the morpholino was achieved by injecting 1 nL at concentrations from 0.25 mM (2 ng/nL) to 0.5 mM (4 ng/nL) to determine the optimum level for microinjection (which was 0.5 mM). An exception to this is the MO concentration used in the tubulogenesis/circulation experiments with Rhodamine–Dextran was 0.25 mM. The Fer rescue experiments were performed by injecting 1 nL of 0.5 mM *fer*-MO1 and control MO. Improper splicing with the *fer* MO was confirmed using PCR to amplify the region spanning exon 9, using a forward primer in exon 8 (5′-AGTCCACCACAGAGGAGCTG-3′) and a reverse primer in exon 10 (5′-AGTCTGTCCTTGGCTCTTCG-3′) (Figure 2I). Although not shown here, experiments repeated with MOs and PCR primers, as reported by Paardekooper Overman, et al. (P-O MO), confirmed specificities for the Fer gene were consistent with data obtained using our *fer*-MO1 (Fer protein knockdown with both MOs is shown in Supplementary Figure S1 as *fer*-MO1 and P-O MO) [20]. Morpholino knockdown was confirmed using a Western blot analysis, using an anti-Fer monoclonal antibody (Cell Signaling).

### 2.3. Whole Mount In Situ Hybridization (WISH)

Embryos were collected and fixed in 4% PFA overnight at 4 °C before performing WISH, as previously described [35]. Probes for *fer*, *fli1*, *gata1*, *scl*, *c-myb*, *hbbe2*, and *runx1* were generated from linearized plasmid containing the cDNA of interest, using either T7 or SP6 RNA polymerase (Ambion) and digoxygenin-UTP labeling mix (Roche). The embryos were washed in a series of glycerol/PBS solutions, where the percent glycerol was increased from 30 to 70 percent, prior to mounting on slides and imaging.

### 2.4. o-Dianisidine Staining

Control and *fer*-MO1 embryos were collected and dechorionated at the desired developmental stages. They were then immediately stained for approximately 30 min in a fresh solution of 0.6 mg/mL o-dianisidine, 0.01 M sodium acetate (pH 4.5), 0.65% H_2_O_2_, and 40% ethanol. After staining, they were cleared with a solution of 2:1 benzyl benzoate/benzyl alcohol. 

### 2.5. Microangiography

At 48 h post-fertilization (hpf), *fer*-MO1 and control embryos (using the *fli1a::nEGFP* transgenic line) were anesthetized and mounted in low-melting agarose. They were then injected in the dorsal aorta (trunk region at the posterior end of the yolk extension) or the sinus venosus with 1 nL of a 12 nM solution of red fluorescent Rhodamine–Dextran beads (70,000 MW, Molecular Probes, Eugene, OR, USA) and given 15–20 min for the beads to circulate prior to imaging.

### 2.6. Quantitative PCR (qPCR)

Using a QIAGEN RNeasy kit, the total RNA of control and *fer*-MO1 injected embryos was isolated at the indicated time points and then reverse transcribed to cDNA using a FirstChoice RLM RACE kit (Ambion), which was used in all subsequent PCR reactions. Primer pairs for *kdrl/flk1*, *fli1*, *gata1*, *scl*, *runx1*, *notch1b*, *c-myb*, *hbbe2* and *fer* were designed using Primer3 and chosen to have fragment size between 150 and 200 base pairs (Supplemental Table S1). qPCR was performed using a SsoFast EvaGreen Supermix (BioRad) and a C1000 thermocycler with the CFX96 Real Time System (BioRad). Experiments were repeated twice, with gene targets analyzed in triplicate with each run. A melt curve was used to confirm the purity of the analyzed product. The analysis was performed by normalizing the target genes to actin.

### 2.7. Fluorescence Activated Cell Sorting (FACS)

Zebrafish embryos that were injected with *fer*-MO1 or control MO were collected at 28 hpf and subjected to cell dissociation and resuspended in PBS, as previously described [36]. Cells were sorted on a BD FACS Caliber flow cytometer (BD Biosciences, San Jose, CA, USA) using FL3 to determine *gata1::dsRed* fluorescence, and FL1 to determine *fli1a::nEGFP* fluorescence. Cells were gated by *nEGFP* or *dsRed* signal using CellQuest Pro to determine the number of *gata1* and *fli1a* expressing cells in each sample. At least 10,000 cells were counted per treatment and the experiment was repeated twice, with error determined via calculation of the standard deviation.

### 2.8. Fer Kinase Inactivating Point Mutations and Notch Rescue Experiments

Point mutations in *fer* were generated separately in the SH2 and tyrosine kinase domains of the zebrafish Fer with a cloned full length cDNA (clone ID:7060149 from Open Biosystems-Dharmacon, Lafayette, CO, USA) using the QuikChange Lightning Site-Directed Mutagenesis kit (Agilent) and the sequence confirmed (Figure 7O). The plasmids were linearized with NotI and capped mRNA (including point mutants, wildtype and anti-sense) was made using the SP6 mMessage mMachine kit (Ambion). Approximately 25 pg of mRNA was co-injected with 0.25 mM MO (*fer*-MO1 or control MO). The zebrafish Notch Intracellular Domain (NICD) construct was obtained (Addgene, Cambridge, MA, USA, 453 pCSMTN5ICD was a gift from Nathan Lawson, plasmid # 22466)) and capped mRNA made using the SP6 mMessage mMachine kit. NICD expression was confirmed by Western blot analysis, using an anti-activated Notch antibody (AbCam).

### 2.9. Imaging

All imaging was performed on either a Nikon SMZ1500 dissecting microscope, an inverted, epifluorescent Zeiss Apotome microscope or a laser scanning confocal microscope (Nikon A1R and Leica SPE). (Nikon, Tokyo, Japan) (Zeiss, Oberkochen, Germany) (Leica, Wetzlar, Germany).

Three dimensional (3D) reconstructions were obtained by mounting embryos at 28 hpf in low melting agarose and these were imaged following stimulation with the 488 nm and 561 nm diode lasers, using a Plan Apo λ 20x objective. Mounted embryos were placed on the stage of the Nikon A1 microscope within an Okolab stage-top incubation system that maintained a temperately controlled environment of 28.5 °C. A NIS Elements (Nikon) device was used to visualize the zebrafish embryos and perform 3D reconstructions (from z-stacks). 

## 3. Results

### 3.1. Fer Kinase Is Expressed during Embryogenesis

**Figure 1:**
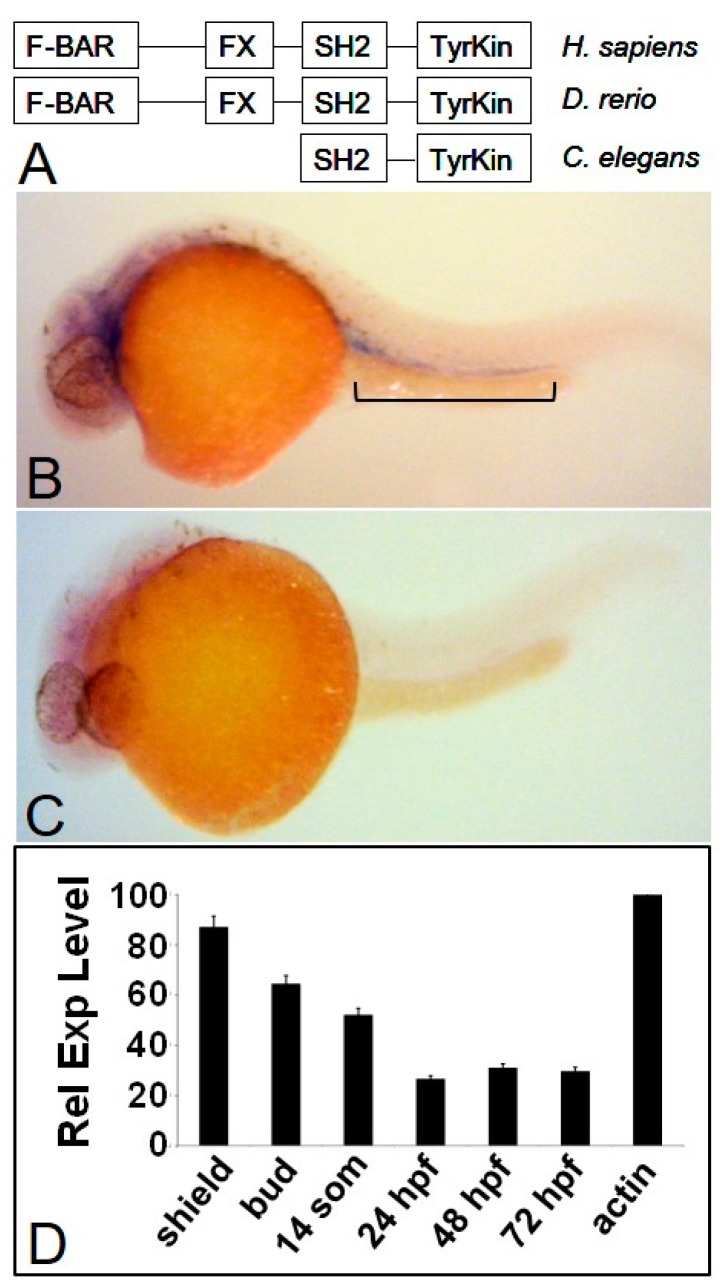
Fer kinase is conserved throughout evolution and is expressed during zebrafish embryogenesis. The zebrafish homologue of Fer kinase is highly conserved throughout eukaryotic organisms and contains the same domains as the human version (**A**). The zebrafish homologue of the *fer* kinase gene is expressed ubiquitously during early developmental stages with more specific expression emerging between 24 and 72 hpf (not shown). At 24 hpf, *fer* expression is observed in the intermediate cell mass (ICM) and developing brain region (**B**), when compared to a *fer* anti-sense probe (**C**). Quantitative PCR shows *fer* expression is highest during early development and tapers off by 24 hpf to three to four-fold lower levels (**D**).

Using the predicted amino acid sequence of the zebrafish Fer, we compared Fer homologues among a wide variety of organisms, ranging from invertebrates to vertebrates (Figure 1A). The resulting phylogenetic analysis showed that the zebrafish Fer is 74 percent homologous to the human Fer, being conserved throughout the F-Bar, FX, SH2 and tyrosine kinase domains, thus serving as an important “bridge” between the invertebrate and vertebrate versions of Fer. We generated gene specific probes to *fer* cDNA for in situ hybridization, to determine the spatio-temporal expression during development. Similar to what was reported by Paardekooper Overman et al. [20], we found *fer* to be expressed ubiquitously until approximately ten somites, when it became more specifically concentrated toward the head region. However, we also observed *fer* expression in the primordial region of the dorsal aorta (DA)/cardinal vein (CV), when compared to a *fer* antisense probe negative control (In Figure 1B,C, respectively—24 hpf is shown). This expression pattern decreased in intensity until approximately 72 hpf, when *fer* expression was no longer detectable in the ICM region. Confirmation by quantitative PCR showed highest expression of *fer* early in development, which decreased until 24 hpf, when it appeared to stabilize at a level three to four-fold lower than the earliest expression (Figure 1D).

### 3.2. Fer Kinase Is Required for Proper Blood and Vasculature Development in Zebrafish 

**Figure 2:**
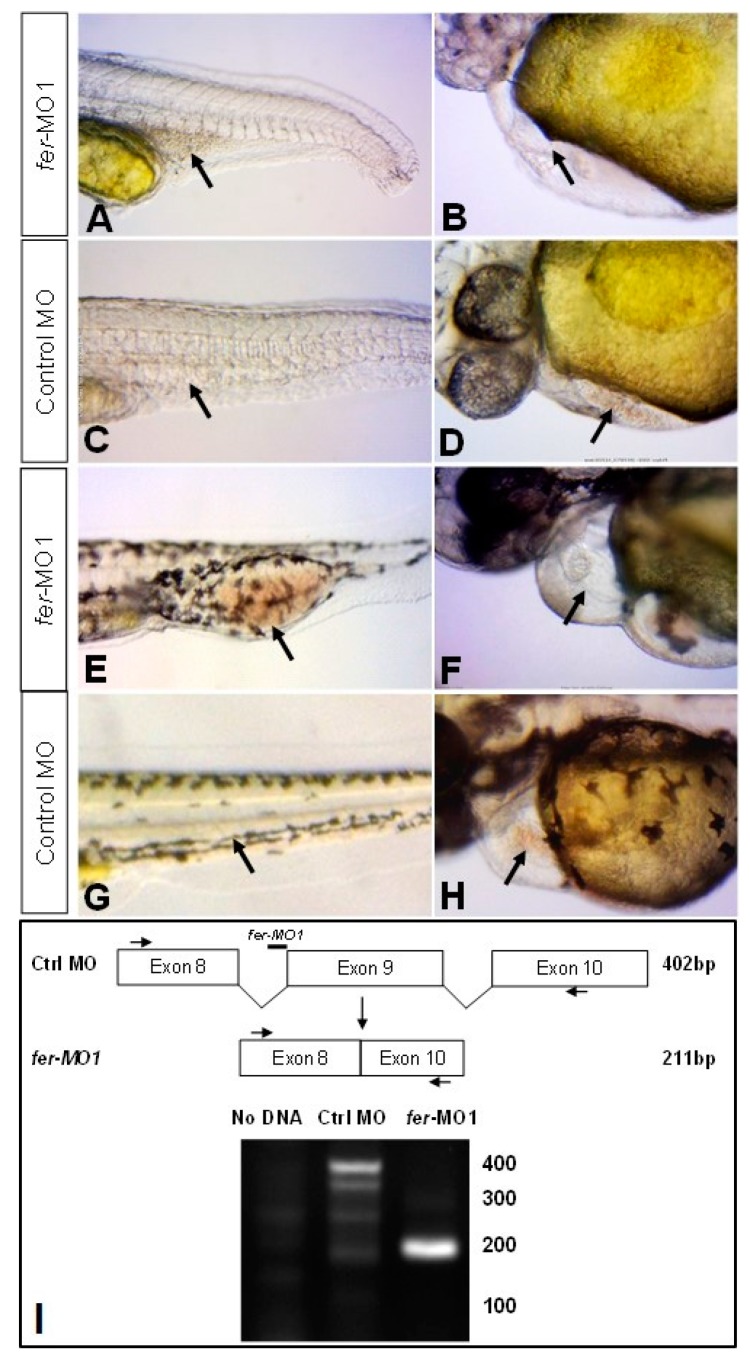
Fer knockdown results in apparent vasculature blockage and accumulation of developing hematopoietic cells. At 30 somites, *fer*-MO1 embryos exhibit convergence extension defects along the anterior–posterior axis with red blood cells that are pooled in the posterior ICM region (**A**) with none present in the pumping heart (**B**), when compared to wildtypes ((**C**,**D**) respectively). By 48 hpf, the lack of circulating red blood cells and empty heart chambers is emphasized in the Fer knockdown embryos (**E**,**F**) compared to wildtype embryos (**G**,**H**). Agarose gel electrophoresis of *fer*-reverse transcription PCR from *fer*-MO1 injected embryos to demonstrate the blockage of Fer splicing at exon 9 (**I**).

The requirement for the zebrafish Fer kinase homologue during development was examined using knockdown with gene specific morpholinos (see methods). In the absence of Fer kinase, we observed the arrest of embryonic development at 72 hpf, with severe defects in hematopoiesis, vasculogenesis, heart development and the central nervous system. In the absence of Fer kinase, at 30 somites (~28 hpf) we observed a lack of circulating blood cells (Figure 2A, Supplemental Video S1) when compared to control MO injected embryos (Figure 2C, Supplemental Video S2); this was emphasized by the absence or pooling of hematopoietic cells in the region of the ICM. All embryos were imaged after reaching the same stage, which, in the absence of Fer kinase, resulted in a shortening of the anterior–posterior axis, similar to what was observed by Paardekooper Overman et al. [20]. In addition to the lack of circulating erythrocytes, the absence of Fer kinase activity led to general cardiac development defects, along with pericardial edema (Figure 2B,F, Supplemental Video S3) when compared to wildtypes (Figure 2D,H, Supplemental Video S4). In Fer deficient embryos, the heart chambers both contracted, although in the majority of embryos, the heart chamber contraction occurred erratically and asynchronously, when compared with wildtypes. In this study we therefore chose to focus our investigation on the hematopoietic and vascular defects.

We found there were two categories of severity in the loss of circulation in *fer*-MO1 injected embryos, with 78.4 percent of embryos containing primarily non-circulating erythrocytes and 21.6 percent of embryos containing no visibly circulating erythrocytes (*n* = 223). A loss of circulating erythrocyte populations was observed in control MO injected embryos less than one percent of the time (*n* = 258). In the category containing non-circulating erythrocytes, the *fer*-MO1 phenotype became more pronounced by 48 hpf (Figure 2E), with the group of non-circulating erythrocytes expanding in number in the region of the ICM, compared to the control MO embryos, where no non-circulating erythrocytes were observed (Figure 2G).

**Figure 3:**
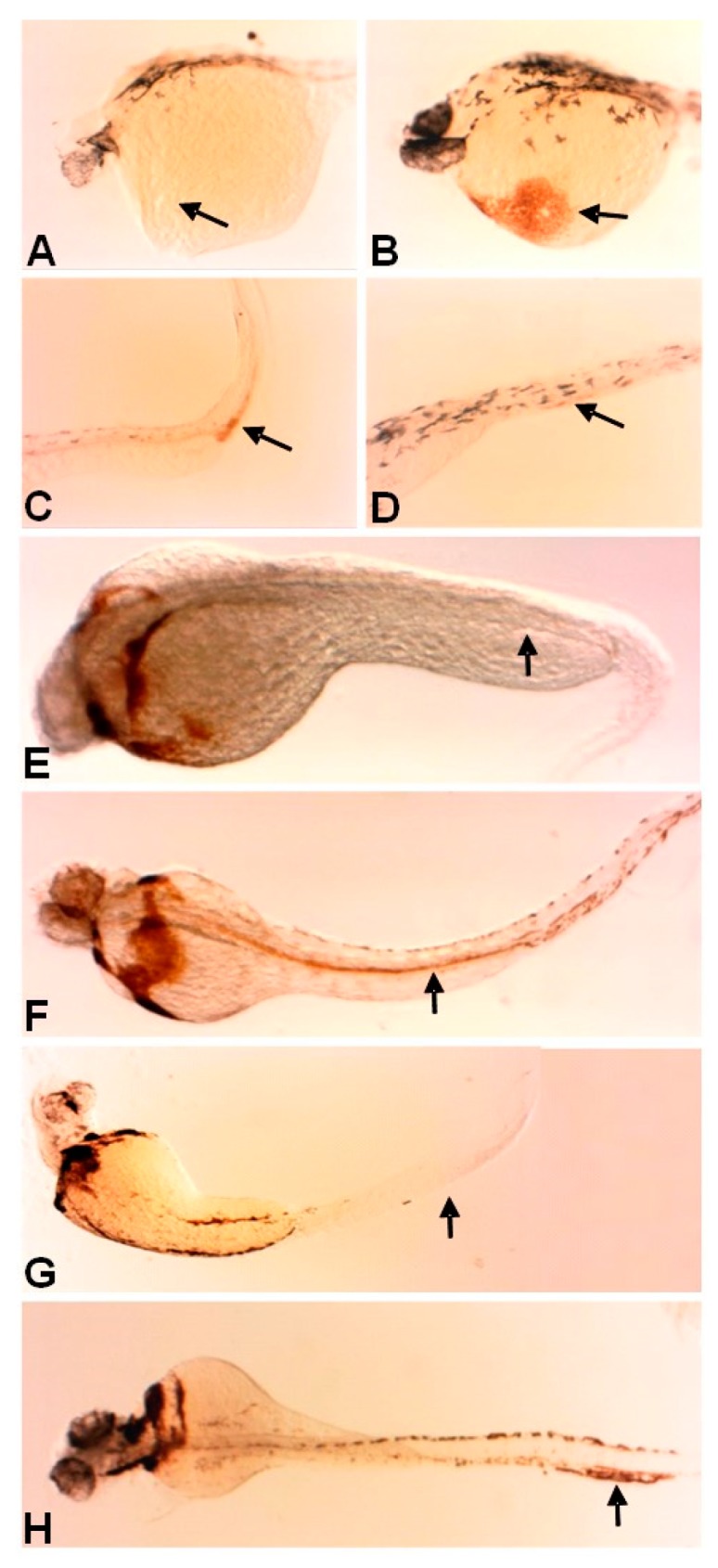
The loss of Fer kinase function resulted in the loss of early anterior erythrocyte formation and the loss of circulation. o-Dianisidine staining showed the slightly higher levels of erythrocyte heme groups in the posterior trunk region of *fer*-MO1 embryos at 30 somites (~28 hpf) (**C**, control-**D**), but not in the anterior (**A**), as is observed in control embryos (**B**). The staining was no longer present in the posterior region at 48 and 72 hpf in *fer*-MO1 ((**E**,**G**) respectively) but regained anterior staining, indicating that later erythrocyte production is functional but no circulation is taking place (control **F** and **H**, 48 and 72 hpf respectively).

To confirm that the cells present were differentiated erythroid cells, we performed o-dianisidine staining, which labels erythrocyte populations expressing hemoglobin. The loss of Fer kinase resulted in decreased erythrocytes in the anterior region and visible pooling of cells in the posterior (ICM) region of the embryo at 28 hpf (Figure 3A,C, control embryos Figure 3B,D, *n* = 25 per condition). At 48 and 72 hpf, erythrocytes in the *fer*-MO1 embryos were present in the anterior region, due to the normal production of erythrocytes that occurs during this time from the aorta-gonad-mesonephros (AGM). However, in the most severe embryos (lacking Fer activity), there was still no detection of hemoglobin containing cells in the posterior trunk region during these time points (Figure 3E,G, *n* = 25 per condition). In contrast, control MO injected embryos contained erythrocytes throughout their entire circulatory system (Figure 3F,H). Normally, by 48 hpf, erythrocytes are no longer produced in the region of the ICM, thus the lack of trunk staining of the *fer*-MO1 embryos at 48 and 72 hpf suggests the absence of circulation rather than an inhibition of erythrocyte development at the later time points.

**Figure 4:**
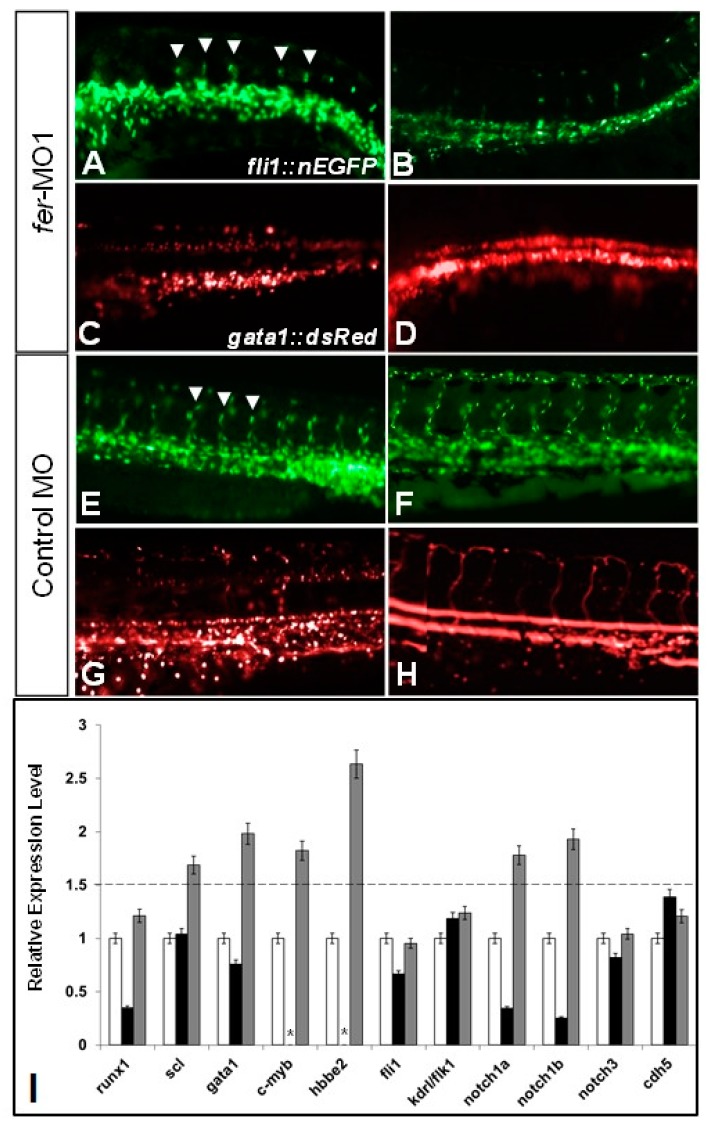
The loss of Fer kinase function resulted in intersegmental vessel (ISV) formation defects and loss of blood flow in zebrafish embryos. *fer*-MO1 injected embryos failed to properly organize the dorsal aorta (DA) and cardinal vein (CV) regions (**A**,**B**), and had fewer ISV structures form (white arrowheads), when compared to the control MO injected embryos (**E**,**F**). Additionally, at similar time points in the absence of Fer kinase, hematopoietic precursor cells formed in the region of the DA but failed to circulate (**C**,**D**) (control MO—(**G**,**H**)). Vasculature was visualized using *fli1::nEGFP* and hematopoietic cells were visualized using *gata1::dsRed*. In (**C**,**D**,**G**,**H**), punctate dots indicate stationary cells, while continuous lines indicate moving cells. Time points are at 30 somites (~28 hpf) for (**A**,**C**,**E**,**G**) and 48 hpf for (**B**,**D**,**F**,**H**). (Note: some cells in appear overexposed due to the abundance of overlapping cells in one region.) The quantitative PCR showed that the expression of hematopoietic precursor genes (*scl*, *gata1*, *notch1a*, *notch1b*, *notch3*, *c-myb*) were increased in the absence of Fer kinase activity (runx1 is decreased early but recovers by 30 somites), while genes expressed in the vascular endothelium remained generally unaffected (*fli1, kdrl/flk1*). The cell–cell adhesion complex component, *cdh5* (VE-cadherin) expression was unaffected in the absence of Fer kinase (**I**). (black bars indicate Fer MO at 8 somites, gray bars indicate Fer MO at 28 somites, * indicate not done). The dotted line denotes the threshold for up-regulated expression, when normalized to the wildtype expression levels, which was set at a value of 1 on the y-axis.

To better visualize the defects in erythrocyte circulation in the absence of Fer kinase we generated a stable *fli1a::nEGFP*, *gata-1::dsRed* double transgenic line to observe both vascular and hematopoietic cells in the same embryos. Using this model, in the absence of Fer activity, we observed a lack of organization within the region forming the dorsal aorta and the cardinal vein, as well as a significant loss of ISV formation (i.e., decrease in the number of *fli1a::nEGFP* migrating cells) at both 30 somites (~28 hpf) and 48 hpf in 82.1 percent of the embryos (*n* = 56) (Figure 4A,B), when compared to the control embryos where no loss of vascular organization was observed in any embryos (*n* = 47) (Figure 4E,F). Additionally, we found that in embryos with disorganized vasculature, there was a corresponding abundance of *gata1::dsRed* expressing cells in the region of the ICM. However, these gata1::dsRed cells were completely stationary, with no observable movement into the ISVs (Figure 4C,D show punctate dots); which is in contrast to the control embryos, where cells could be observed moving within the dorsal aorta (Figure 4G,H—static images, lack of puncta in H indicates circulating cells).

**Figure 5:**
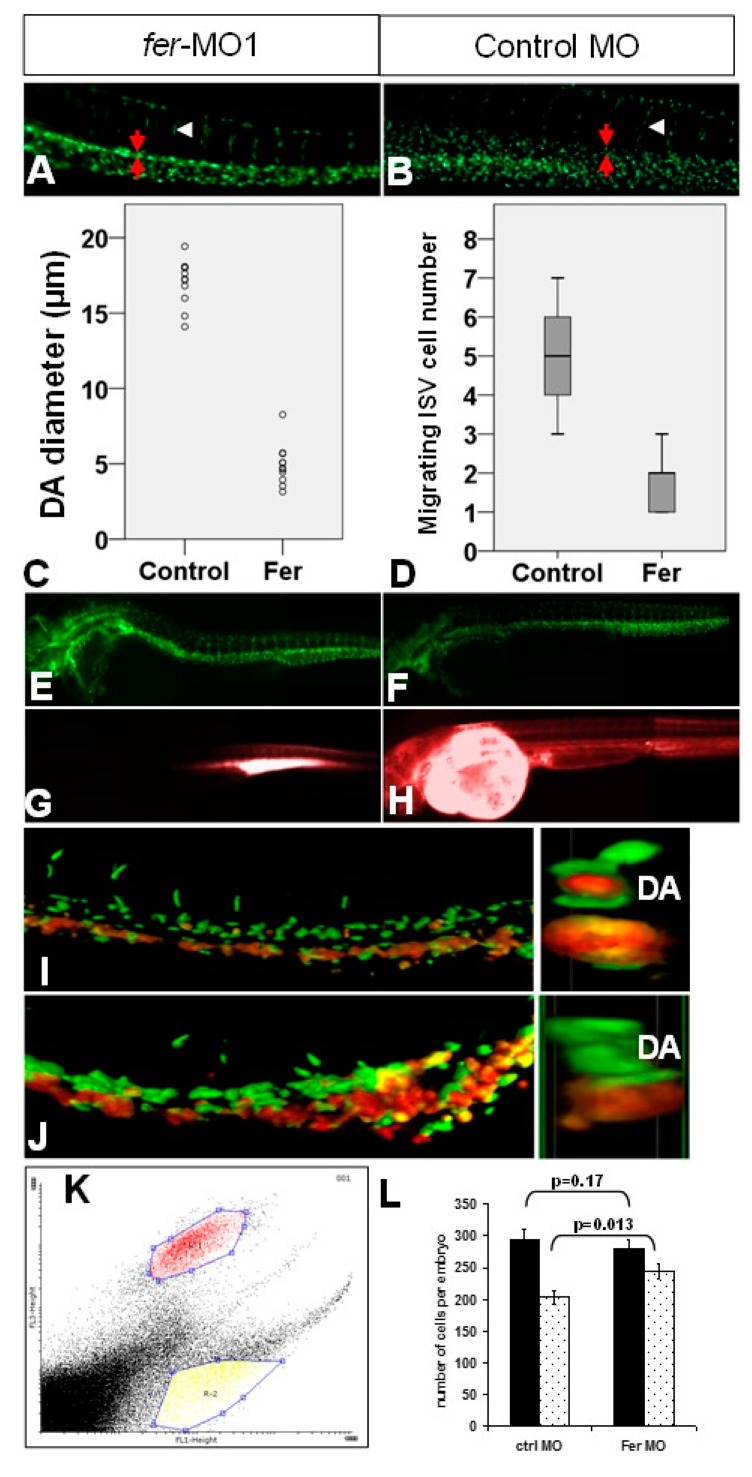
The loss of Fer kinase function resulted in vascular tubulogenesis defects. The loss of Fer kinase function decreased the diameter of the dorsal aorta (**A**,**C**) and resulted in fewer migrating cells into the ISV regions (**D**) at 30 somites (Control embryos (**B**–**D**). Rhodamine–Dextran injections into DA resulted in no visible dye circulation in Fer deficient embryos ((**E**), *fli1::nEGFP*, (**G**), Rhodamine), when compared to control embryos ((**F**), *fli1::nEGFP*, (**H**), Rhodamine). Confocal microscopy images demonstrated a concurrent loss of vasculature organization in the region of the DA and gain of hematopoietic precursors at 26 hpf ((**I**), control MO, (**J**), *fer*-MO1). The right images in (**I**,**J**) are digital cross-sections, taken approximately midway through the image; z-stacks showed no gata1::dsRed signal in the region that should be the inner-DA. The FACS analysis showed a shift in the number of *gata1::dsRed* cells, along with a loss of vascular endothelial population ((**K**), gated regions of non-overlapping, *fli1a::nEGFP* and *gata1::dsRed* cells, (**L**), quantitative plot showing differences in the cell population from (**K**) in the presence/absence of Fer kinase activity).

We then tested the hypothesis that the lack of circulating erythrocytes in the absence of Fer was due primarily to defective vasculogenesis. By measuring different aspects of the trunk vasculature, we determined that in *fer*-MO1 embryos, at 30 somites, there was a significant decrease in the diameter of the dorsal aorta (DA) (Figure 5A, red arrows) and a loss of migrating cells into the intersegmental vessel (ISV) region (Figure 5A, white arrowhead), when compared to control MO embryos at the same stage (Figure 5B). Embryos lacking Fer kinase activity had a mean DA diameter of 4.93 μm, compared to 17.03 μm for control MO injected embryos (Figure 5C, *n* = 7 per treatment, *p* = 1.91e−14). Additionally, the number of cells migrating from the dorsal aorta into the region of the intersomitic boundaries was 1.78 cells per ISV in *fer*-MO1 embryos, less than half as many as the control MO embryos, which had 4.86 cells per ISV (Figure 5D, *n* = 25 per treatment, *p* = 1.42e−34).

With a significantly smaller DA diameter and fewer cells migrating into the intersegmental boundaries, we then asked whether the vasculature that did form in the trunk region was undergoing tubulogenesis. We performed injections of a Rhodamine–Dextran conjugate into the dorsal aorta and sinus venosus regions of morpholino injected embryos (*fer* and control). With the severity of the disorganized posterior trunk vasculature using 0.5 mM *fer*-MO1, we titrated the MO concentration and determined that at 1 nL of 0.25 mM MO we were able to observe a higher level of organization, with a larger DA and more cells migrating in the intersegmental boundary regions (Figure 5E, control MO—Figure 5F). Thus, we used this lower concentration of MO to determine whether, even in the presence of increased normal vasculature organization, there was proper tube formation in the absence of Fer kinase. In control MO injected embryos, we observed normal distribution of the Rhodamine dye within fifteen minutes post-injection (*n* = 12) (Figure 5H). However, in the *fer*-MO1 injected embryos, the dye never migrated beyond the area of injection, at up to one hour post-injection (*n* = 9) (Figure 5G). Our results suggest that, even in the presence of vasculature and a beating heart, there is not a sufficient amount of organized, open tubes within the trunk endothelium, to allow for proper circulation of existing blood cells in the absence of Fer kinase function. (sinus venosus injection data resulted in similar loss of dye circulation [37].

Using confocal microscopy we were able to support our previously observed phenotype by showing that in the same embryo, the absence of Fer caused both defective DA/CV remodeling and ISV cell migration (*fli1a::nEGFP*), as well as increased populations of gata1::dsRed expressing cells in the DA (Figure 5J, control embryo—Figure 5I) (*n* = 10 per condition). By analyzing the confocal images in a cross section, we were able to show that *fer*-MO1 embryos lacked *gata1::dsRed* cells migrating through what is normally the lumen of the DA, in contrast to control MO injected embryos (Figure 5J,I respectively, right panel). This data further supports the proposal that there are no circulating hematopoietic cells due to improper tubulogenesis within the trunk vasculature.

Our data, showing either a loss of hemoglobin expressing erythrocytes, or a pooling of existing erythrocytes, in addition to a lack of vascular organization, caused us to question whether there was a shift in the number of cells within these populations in the absence of Fer. We tested this by performing a fluorescence activated cell sorting (FACS) analysis with embryos containing both the *fli1a::nEGFP* and *gata1::dsRed* expressing cells. *fer*-MO1 embryos were separated into the two categories of severity described earlier, where one category of embryos had a pooling of hematopoietic cells and another, more severe category contained no observable hematopoietic cells under white light microscopy. Since the majority of embryos contained the pooled hematopoietic cells, we chose to use only those for the FACS analysis.

To clarify the fluorescent populations, the gated regions, represented in Figure 4K, demonstrate that we counted cell populations that reflected the expression of either *fli1a* (nEGFP) or *gata1* (dsRed). Since these are live cells, we could not avoid the fact that dsRed goes through an intermediate state that fluoresces in the nEGFP spectrum while folding, and thus, appears to be both nEGFP and dsRed positive [38,39]. However, because the cells contain a combination of dsRed and GFP-like fluorescence, we were able to distinguish it from the *fli-1a::nEGFP* only population (Figure 5K). When we tested injected embryos using a FACS analysis of the gated regions, the *fer*-MO1 embryos at 30 somites showed a significant increase in the number of gata1::dsRed positive cells per embryo, from 203 in the control embryos, to 244 in the *fer*-MO1 embryos (*p* = 0.013) and, conversely, no effective change in the number of *fli1a::nEGFP*, from 294 cells per embryo in the control embryos, to 280 cells per embryo in the absence of Fer (*p* = 0.17) (Figure 5L). Although this increase in cell number appears moderate, it is reasonable based on previous studies examining the conversion of vascular precursors in the dorsal aorta into erythrocytes [40]. Thus, we further investigated whether the endothelial cells are converted to erythrocytes or there is excess proliferation within the erythrocyte population.

**Figure 6:**
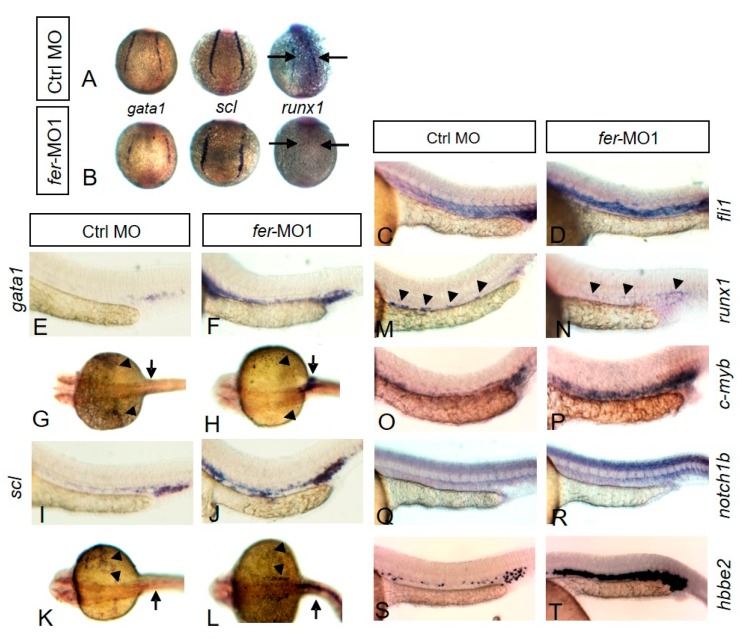
Fer kinase is required for proper expression patterns of hematopoietic genes. In situ hybridization showed the expression of gata1, scl and runx1 at the 6 somite stage in the region of the lateral plate mesoderm. Control embryos are shown in (**A**) while *fer*-MO1 embryos are shown in (**B**). Arrows point to the region of runx1 expression. At 30 somites, the expressions of *fli1* (**C**,**D**), *gata1* (**E**–**H**), *scl* (**I**–**L**), *runx1* (**M**,**N**), *c-myb* (**O**,**P**), *notch1b* (**Q**,**R**), and *hbbe2* (**S**,**T**) are shown.

With a distinct link between the loss of differentiated erythrocytes and lack of proper vasculogenesis, we next sought to determine whether there were gene expression changes in the early genes that specify the direction of differentiation beyond the hemangioblast cell populations early in development. We examined the expression of erythrocyte determining genes at the 6 somite stage of all embryos and found that *gata1* and *scl*, both normally expressed in the lateral plate mesoderm, appear to have slightly decreased levels and a discontinuous pattern in the *fer*-MO1 embryos (Figure 6B), when compared to control MO embryos (Figure 6A) (*n* = 20/26 and 25/32 respectively). Although the shape of the embryos appears wider, due to the convergent extension defects that lead to a shortening along the anterior–posterior axis of the embryo, the overall expression levels of the *gata1* and *scl* genes appear to be mostly intact.

However, when we examined expression of runx1, shown to be required for proper erythrocyte development and vasculogenesis [41], we observed a severe decrease in the expression of the posterior lateral plate mesoderm in the absence of Fer (*n* = 23/36) (Figure 6B) when compared to control embryos (Figure 6A—black arrows).

Due to the altered nature in the expression of *gata1*, *scl* and *runx1* earlier, we then examined the later expression of genes involved in both vasculogenesis and hematopoiesis in the region of the ICM. At 30 somites, the vascular endothelium marker, fli1, was expressed at normal levels but showed that in 79.1 percent of *fer*-MO1 embryos there was a general lack of organization in the trunk region, with no observable structural differentiation within the DA and CV regions, as well as no migrating ISV cells (*n* = 34/43) (Figure 6D), when compared to control embryos at the same stage (*n* = 30) (Figure 6C).

Probes against hematopoietic genes, such as *gata1* (*n* = 44/52) (Figure 6F,H), *scl* (*n* = 47/55) (Figure 6J, L), *c-myb* (*n* = 34/41) (Figure 6P) and *hbbe2* (*n* = 41/48) (Figure 6T) stained a larger population of cells in the absence of Fer kinase, with the region of cells expressing these markers expanded in the anterior direction (control MO is shown in Figure 6E,G (*gata1*), I,K (*scl*),O (*c-myb*),S (*hbbe2*) respectively), suggesting either an up-regulation of expression with these genes or an increase in the number of cells expressing these genes. We also observed a partial recovery of runx1 expression (*n* = 30/38) (Figure 6N), when compared to the control embryos (*n* = 40) (Figure 6M). We next examined each of these genes using quantitative PCR and confirmed an increase in expression correlating to that observed with our in situ hybridization analyses, with increases in expression, ranging from 1.5 to 2.5 fold, over control embryos at the same stage of development (Figure 4I). One notable exception was that of runx1, which appears to be expressed at normal levels, which again, is in agreement with the in situ hybridization results we observed.

The lack of migrating ISV cells during development suggested to us that there may be aberrant Notch function in the absence of Fer, as Notch has been well known to regulate the formation of ISVs [42,43]. Initially, we thought that perhaps an overexpression of a Notch signal might be responsible for the defects related to the absence of Fer. We then investigated the expression levels of *notch1a*, *notch1b* and *notch3* during development in *fer*-MO1 embryos at two time points (8 somites and 28 somites) using quantitative PCR (Figure 4I). Surprisingly, notch3 expression was not altered; however, levels of both *notch1a* and *notch1b* were both decreased by more than half the normal levels at 8 somites, and elevated in excess of 1.5 fold at 28 somites. Thus, we examined *notch1b* via WISH and observed that, consistent with our qPCR data, it was indeed up-regulated in the absence of Fer, when compared to control MO embryos, in the trunk region (Figure 6R,Q, respectively) and the head [37]. 

### 3.3. Fer Kinase-Dependent and -Independent Activity during Development

**Figure 7:**
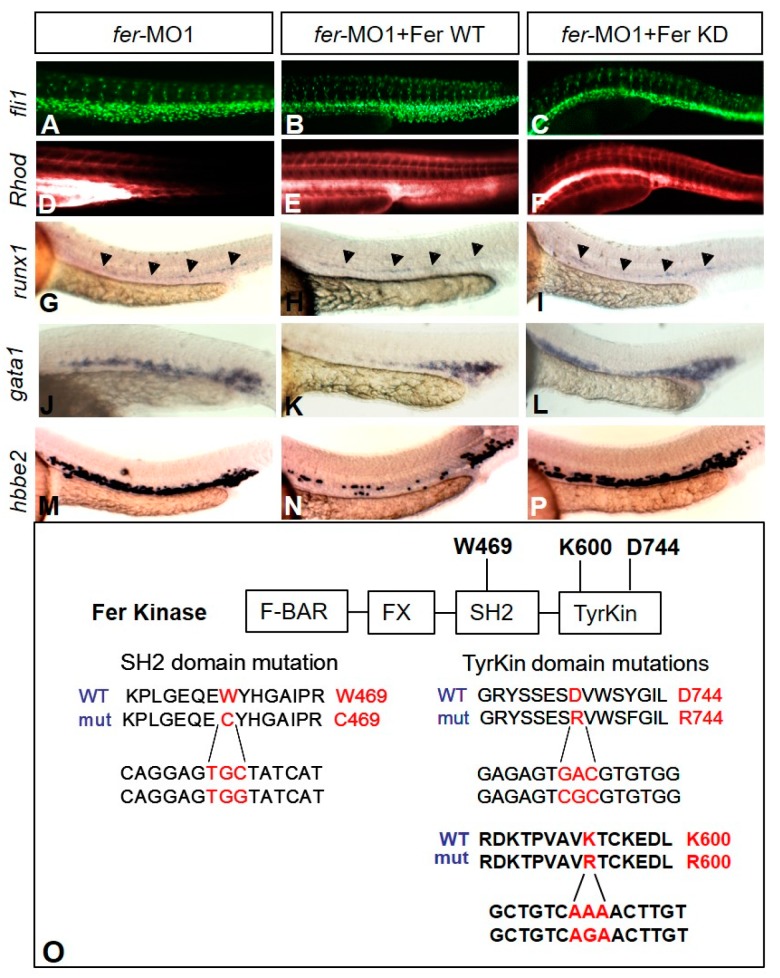
Kinase-inactive Fer rescues vascular tubulogenesis but not hematopoiesis gene expression defects. The injection of *fer* mRNA and *fer*-MO1 into *fli1a::nEGFP* embryos results in increased cell migration into the ISV regions ((**B**)—WT, (**C**)—kinase inactive (KD)) when compared to *fer*-MO1 alone (**A**), and rescues tube formation ((**E**)—WT, (**F**)—KD, Rhodamine-Dextran circulation after 15 min, when compared to *fer*-MO1 alone (**D**)). Wild type Fer ameliorates defects in erythrocyte expansion, as observed with runx1 (**H**), *gata1* (**K**) and *hbbe2* (**N**), while inactive Fer does not ((**I**,**L**,**P**) respectively). *fer*-MO1 alone is shown in (**G**,**J**,**M**) respectively. Embryos were examined at 30 somites (~28 hpf). Domains of Fer kinase in zebrafish, showing the highly conserved tryptophan and aspartic acid residues, in the SH2 and tyrosine kinase domains, respectively, are shown. Mutating either tryptophan to cysteine (W469C), aspartic acid to arginine (D744R) or lysine to arginine (K600R) resulted in the loss of kinase activity for Fer. The amino acid sequence is shown on top, with the corresponding DNA sequence changes below ((**O**), Fer KD expression confirmation shown in Supplementary Figure S1).

To further investigate how Fer kinase functions during development, we tested whether the kinase activity of Fer was required in specifying endothelium and hematopoietic cells. We generated two separate point mutations that have previously been shown to effectively inactivate the kinase activity of Fer in mice (SH2 and TyrKin mutations) and worms (TyrKin mutation) [12,16]. The first point mutation was in the SH2 domain and changed a tryptophan to a cysteine, while the second and third mutations converted an aspartic acid to an arginine, and lysine to arginine, respectively, in the tyrosine kinase domain (Figure 7O, Western blot confirmation of protein expression shown in Supplementary Figure S1). We then attempted to rescue the *fer*-deficient phenotypes by co-injecting *fer*-MO1 (0.25 mM) with *fer* mRNA (wildtypes or containing either point mutation). It is important to note that we used a cloned cDNA for *fer*, thus eliminating the possibility that the mRNA might be targeted by the splice blocking MO (which exclusively targets an intron sequence). In three independent experiments, wildtype *fer* mRNA was able to rescue both the vascular and hematopoietic defects in the absence of endogenous Fer kinase activity, when compared to a control co-injection of *fer*-MO1 and *fer* anti-sense mRNA (Supplementary Figure S2). The DA and ISV organization allowed for proper tubulogenesis to take place, as injection with the rhodamine–dextran dye showed circulation throughout the embryo (*n* = 9) (Figure 7B,E) compared to *fer*-MO1 alone (Figure 7A,D). Additionally, in situ hybridization demonstrated that expression of hematopoietic directing genes such as *runx1* (*n* = 29/33), *gata1* (*n* = 32/37), and *hbbe2* (*n* = 47/52) were restored to normal spatio–temporal expression patterns (Figure 7H,K,N), when compared to *fer*-MO1 alone (Figure 7G,J,M).

Interestingly, both point mutants were able to rescue tubulogenesis within the vasculature, with circulation of the Rhodamine-Dextran dye evident within fifteen minutes of injection (*n* = 12) (Figure 7C,F—TyrKin (D744R) point mutant results shown only). However, neither of the Fer kinase inactive point mutants (denoted as Fer KD) were able to rescue the hematopoietic cell population defects, as evidenced by the fact that there was no significant difference between the *fer*-MO1 embryos and the *fer*-MO1/*fer* mRNA (WT or KD) injected embryos, with respect to expanded expression of *runx1* (*n* = 25/31), *gata1* (*n* = 23/28) and *hbbe2* (*n* = 41/45) (Figure 7I,L,P). These data strongly suggest that there are both kinase-dependent and independent functions to Fer that are required during normal development in zebrafish. Specifically, we found that the Fer kinase-dependent activity was associated with the expansion of erythrocyte gene expression, while the kinase-independent properties of Fer were required in the organization of the vasculature.

### 3.4. Fer Stabilizes Notch Signaling during Vasculogenesis but Not Hematopoiesis

Finally, due to the established links with Notch signaling, regulating both hematopoietic and angiogenic signaling in zebrafish [44], along with our data showing increased expression of the *notch1b* gene in the absence of Fer, we hypothesized that Fer functions in regulating Notch signaling during vasculogenesis and hematopoiesis. It has been shown that increased Notch activity suppresses migration of endothelial cells into the ISV regions from the DA [45], as well as facilitates expansion of the growing erythrocyte populations [46]. Additionally, previous reports have demonstrated that the absence of Fer destabilizes cadherin complexes, and thus, cell–cell adhesions [16,21,24], which would have a downstream effect on juxtacrine signaling. Knowing this, we tested our hypothesis that, although we observed increased notch1 expression, a portion of the phenotype in the absence of Fer could be due to the prevention of juxtacrine mediated Notch signaling as a potential consequence of lost cell-cell adhesion.

**Figure 8:**
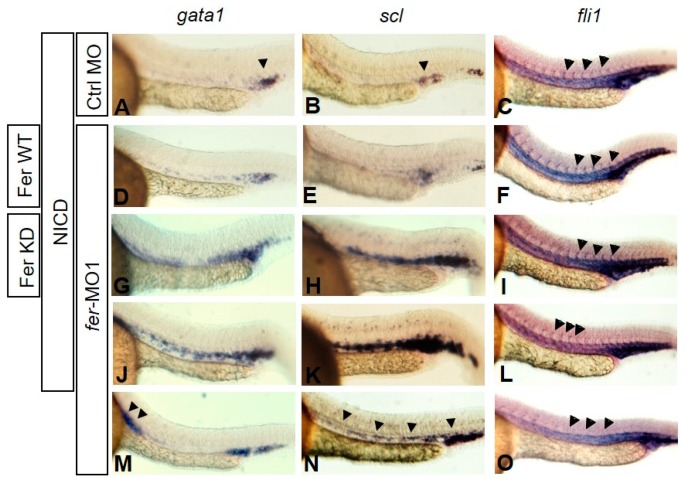
Fer functions independently of Notch for erythrocyte population expansion but not intersomitic vessel formation. Co-injection of the Notch Intracellular Domain (NICD), along with wildtype (WT) or kinase-inactive (KD) Fer, showed that active Notch partially rescues vascular migration in the absence of Fer (*fli1*: (**F**)—WT, (**I**)—KD), but not excess erythrocyte proliferation (*gata1*: (**D**), WT, (**G**), KD; scl: (**E**), WT, (**H**), KD), when compared to fer-MO1 embryos containing only the NICD (**J**,**K**,**L**). Control MO embryos with injected NICD show normal gene expression of *gata1* (**A**), *scl* (**B**) and *fli1* (**C**). *fer*-MO1 alone—*gata1* (**M**), *scl* (**N**) and *fli1* (**O**). Embryos examined at 30 somites (~28 hpf).

Thus, we attempted to rescue the *fer*-MO1 phenotype, by activating Notch, regardless of cell–cell adhesion. We co-injected mRNA expressing the activated form of Notch (NICD—Notch Intra-Cellular Domain) along with mRNA expressing either wildtype or kinase inactive Fer (WT or KD) (NICD expression confirmed via Western analysis is shown in Supplementary Figure S1). After titrating amounts of both NICD and *fer* mRNA, to eliminate non-specific, toxic effects, we found that injecting 10–15 pg of NICD mRNA with the control MO (0.5 mM) had no effect on the development of hematopoietic cells or vasculogenesis (*n* = 52) (Figure 8A–C, respectively), as did injection with the control MO alone (data not shown). Knocking down Fer function resulted in an expansion of hematopoietic markers such as *gata1* and *scl* (Figure 8M,N, respectively). Additionally, reduced Fer function also inhibited ISV sprouting, as observed with fli1 expression (Figure 8O, arrowheads). However, when co-injected with the *fer*-MO1 (0.5 mM), the activated form of Notch was able to partially rescue vasculogenesis, as measured by the presence of fli1+ cells migrating into the ISV region (58.7% rescue, *n* = 63) (Figure 8L—arrowheads), but had no effect on the hematopoietic gene expression defect (87.2% defective, *n* = 55) (Figure 8J,K), suggesting that expansion of hematopoietic cells requires Fer kinase activity, in addition to activated Notch. The ability of NICD to rescue vascular endothelial cell organization was further enhanced when co-injected with the wildtype version of Fer (82.7% rescue, *n* = 58) (Figure 8F), and was also sufficient to rescue the hematopoietic gene expression defect (86.9% rescue, *n* = 61) (Figure 8D,E). Surprisingly, the NICD was also able to rescue the Fer deficient vasculogenesis defect when co-injected with the kinase-inactive Fer (Figure 8I) almost as effectively as with the wildtype Fer (76.6% rescue, *n* = 47), but not the expanded hematopoietic gene expression (79.1% defective, *n* = 53) (Figure 8G,H). We were not able to determine whether the erythrocyte gene expansion was primarily due to up-regulated Notch mediated specification or hyperactivated proliferation. However, these results suggest that, in contrast to hematopoietic populations, the vasculogenic organization events, via Notch activation, are either enhanced, or stabilized, in the presence of Fer, regardless of Fer kinase activity.

## 4. Discussion

The challenges in uncovering functions of Fer kinase during vertebrate development have remained somewhat elusive, due to issues with obtaining a genomic knockout of the gene. Although evidence has been reported as to the function of Fer kinase during mouse development, much of the data has been obtained using a kinase inactive knock-in version of the gene, which results in viable mice, implying Fer may serve mainly in redundant roles during development [12]. We have observed that in the absence of Fer kinase in zebrafish, embryonic development is arrested by 72 h post fertilization, in agreement with what was previously reported by Paardekooper Overman, et al. [20]. Here we reported a novel defect with vasculogenesis and erythrocyte differentiation in the absence of Fer kinase. Additionally, we demonstrated the presence of kinase-independent activity of Fer, which may partially explain the viability of the knock-in, kinase inactive Fer mouse [12]. Furthermore, similar to our results, under normal conditions, Fer kinase expression has been observed in hematopoietic precursors in mice [4]. The Fes/Fps kinase has been shown to redundantly regulate hematopoiesis, along with Fer, through Stat5 and Erk [47]; however, the in vivo role of Fer in the differentiation and proliferation of this cell type has not been previously characterized in developing vertebrate embryos.

The role of Runx1 in zebrafish development has been well characterized, with the mutant being initially reported to prevent both definitive hematopoiesis and vessel development [41], and more recently runx1 was shown to be expressed in early hemogenic endothelial cells [48]. That study showed a loss of circulation, due to improper vessel growth and an accumulation of erythrocyte precursors. This is similar to what we observed in our experiments knocking down Fer kinase activity. It has also been reported that Runx1 is tyrosine phosphorylated by Src family kinases in a manner that is required for proper hematopoiesis to occur [49]. The similar phenotypes, linked by our data, showing an early decrease, followed by a recovery of runx1 expression, suggest that there is a finely tuned regulation of Runx1 function during development, possibly at the level of phosphorylation, if not by gene expression levels.

Our experiments suggest that, in the absence of Fer, there is a defect in the Notch/Runx1 pathway, which results in an increase in *gata1* and *c-myb* expression, and a concomitant increase in erythrocytes. This finding is supported by data in which Cai et al. reported that an AML (Runx1) haploinsufficent mouse model resulted in the early emergence of hematopoietic stem cells [50]. Furthermore, a conditional Runx1 deletion in mice resulted in the depletion of later emerging, adult hematopoietic stem cell populations [51]. Since Notch activates Runx1, it could be that, in the absence of Fer, the normal ratio of hematopoietic and endothelial precursors in later embryos is shifted, in this case due to a hyperactive Notch signal that enhanced the number of erythrocytes at the expense of endothelium prior to the onset of definitive hematopoiesis, a model suggested by [52]. However, our results show that there is an increase in the erythrocyte gene expression without a corresponding major decrease in vascular endothelial cells, similar to the increase in hematopoietic stem cell marker production with the overexpression of the NICD, reported by Burns et al. [44].

Although we could not conclude whether it was expanded, the determination versus proliferation of erythrocyte precursors in the absence of Fer, supported a cellular proliferation role, rather than specification, as it has been previously shown in that Fer kinase is required for proper cell cycle control in models ranging from nematode endoderm proliferation and seam cell division [26,27] to acute myeloid leukemia cell lines [53]. Thus, it is possible that under normal conditions during development, Fer plays a role in regulating the expansion of the trunk hematopoietic cells via Notch signaling.

In the absence of Fer kinase activity, we observed that proper heart development was arrested with the presence of non-circulating erythrocytes, in both heart chambers, along with pericardial edema (Figure 2B–H), which is similar to what was reported by Mitchell, et al. with the knockdown of VE–cadherin in zebrafish [54]. However, since our data showed that the knockdown of Fer kinase caused severe defects in the trunk vasculature, which was not observed with the knockdown of VE–cadherin in Mitchell, et al., it is likely that the *fer*-MO1 defect is not through decreased cadherin expression (Figure 4I), but in the destabilization of cadherin-mediated adhesion complexes. A critical role for Fer in stabilizing cadherin complexes during development has been previously shown with Fer homologues in both nematodes and flies [16,24]. In parallel with cadherin complex stability, the defect we observed could also be due to a migration defect that is dependent on integrin complexes between the plasma membrane and the extracellular matrix used by migrating ISV cells as they move along the intersomitic boundaries. In fact, it has been reported that both Fes/Fps kinase family members and integrin linked kinases (ILK) regulate survival and migration in vascular endothelium, during mouse and zebrafish development, respectively [55,56]. Thus, it is reasonable to hypothesize that Fer kinase mediates activity in the switch from adhesion to migration, as it has previously been shown to engage in cross-talk between cadherin and integrin complexes [33,57].

Although we observed an increase in notch1 expression, endogenous Notch activity may be abrogated by a loss of cell–cell adhesion, as occurs with the loss of cadherin complex stability in the absence of Fer [16,24], which would disrupt juxtacrine dependent processes. It is also possible that the loss of hematopoietic cells that we observed with Fer knockdown is due to blockage of circulatory flow, such as has been reported by North et al. [58]. Alternatively, it could be that Fer functions, in part, to regulate the emergence of hematopoietic gene expression, which would be consistent with the expression of *notch1b* and emergence of HSCs, reported by Kim et al. [59], and that even in the absence of flow, those genes can be activated. However, the vasculogenesis defects we observed in Fer deficient embryos are similar to that reported in the absence of gridlock, a gene downstream of Notch that is required for proper vasculogenesis during zebrafish development [43]. Furthermore, Notch signaling normally acts to regulate angiogenic behavior, such that when Notch activity is lost, there is excess proliferation and migration within the endothelial cell lineage [45,60]. More recently, it was shown that *notch1b* promotes arterial cell fate and prevents tip cell fate, thus sprouting into the ISV from the DA [61]. Our observation that in the absence of Fer kinase, there was a lack of endothelial cell migration out of the DA, combined with an overexpression of *notch1b*, suggests a role for Fer in regulating vascular cell migration. Two likely possibilities in which the absence of Fer would allow the overexpression of Notch in the DA are: (1) suppressed endothelial fates, thereby facilitating a switch to hematopoietic fate, or (2) excess Notch simply prevents initial migration into the ISVs and the hematopoietic expansion occurs in a distinct manner, separate from endothelial cell migration. Any migrating cells moving into the ISVs, however, would likely not be stabilized, due to a potential loss of cell–cell contacts (normally stabilized via Fer).

Finally, the emergence of kinase inactive roles for many protein kinases has become an intriguing topic in recent years. In the case of Fer, the finding that a kinase inactivating knock-in allows sufficient function for development and viability in mice suggests kinase-independent roles for Fer [12]. Although it is possible that defects observed in the absence of Fer are primarily due to the loss of cell adhesion, combined with up-regulation of Notch1, our data also suggest kinase-dependent and independent roles of Fer, which are distinct along the lines of erythrocyte expansion and endothelial adhesion/migration. We suggest a kinase active role for Fer that regulates expansion of erythrocyte precursors, which is enhanced in the presence of activated Notch. Additionally, a kinase inactive function of Fer is necessary in organizing the vasculature, by regulating the Notch signaling that facilitates migrating ISV cells.

The concept of a balance in Fer kinase activity has been supported in other studies, such as over-expression data in metastatic breast cancer and gain of function mutation data from fruit flies [24,62]. In both cases, excess Fer kinase activity destabilized cell–cell adhesion and promoted a disorganized migration of cells, suggesting there is an optimum range of Fer activity that is critical to proper cell function. However, neither study examined the possibility that Fer may function, at least partially, in a kinase-independent manner. This additional function was later shown to be present in both mice [12] and nematodes [16].

## 5. Conclusions

Taken together, our results suggest that Fer kinase activity regulates a finely tuned balance that, when lost, results in an increase in the erythrocyte population, while at the same time, exerts a kinase independent function that facilitates cellular organization in the vascular endothelium. This function of Fer is likely through stabilization of adhesion complexes at the plasma membrane, such as cadherin and integrin complexes, which mediates critical juxtacrine signaling mechanisms. Future studies will likely identify the protein interactions for Fer, which will further clarify the phosphorylation events in proliferation activity as well as kinase-independent, direct interactions facilitating cell–cell adhesion in the regulation of juxtacrine mechanisms, such as the Notch pathway, in vascular endothelial cell migration.

## Supplementary Materials

The following are available online at www.mdpi.com/2079-7737/6/4/40/s1, Figure S1: Fer protein expression analysis; Figure S2: Control MO+*fer* anti-sense mRNA rescue; Table S1: qPCR oligos; Video S1: Fer Kinase MO-1—circulating blood cells; Video S2: Fer Kinase control MO—circulating blood cells; Video S3: Fer Kinase MO-1—arrest of proper heart looping along with pericardial edema; Video S4: Fer Kinase control MO—arrest of proper heart looping along with pericardial edema.
